# Occurrence and Health Risk Assessment of Sulfonamide Antibiotics in Different Freshwater Fish in Northeast China

**DOI:** 10.3390/toxics11100835

**Published:** 2023-10-02

**Authors:** Mengnan Shen, Bowen Yu, Yi Hu, Zhi Liu, Ke Zhao, Chenyang Li, Ming Li, Chen Lyu, Hai Lu, Shuang Zhong, Jie Cheng

**Affiliations:** 1 Key Laboratory of Songliao Aquatic Environment, Ministry of Education, Jilin Jianzhu University, Changchun 130118, China; smn930@aliyun.com (M.S.); catsayer_y@126.com (B.Y.); 13527431798@163.com (Y.H.); chinalz1031@163.com (Z.L.); zhaoke326@126.com (K.Z.); lichenyang0331@126.com (C.L.); mgvi@163.com (M.L.); 2 Key Laboratory of Groundwater Resources and Environment, Ministry of Education, Jilin University, Changchun 130021, China; zhongshuang@jlu.edu.cn; 3 Second Institute of Oceanography, Ministry of Natural Resources, Hangzhou 310012, China; chengjie@sio.org.cn

**Keywords:** bioaccumulation, antibiotic residues, risk assessment, HPLC–MS/MS, food safety

## Abstract

This study aimed to investigate the levels of 12 sulfonamide antibiotics in freshwater fish species obtained from three cities in northeastern China (Harbin, Changchun, and Shenyang). The analysis was conducted using HPLC–MS/MS to accurately quantify the antibiotic concentrations in the fish samples. The results showed that the average levels of sulfonamide antibiotics in fish samples from Harbin, Changchun, and Shenyang were 1.83 ng/g ww, 0.98 ng/g ww, and 1.60 ng/g ww, respectively. Sulfamethoxazole displayed the highest levels and detection rates in all three cities, whereas sulphapyridine exhibited the lowest concentrations in all the fish samples. The levels of sulfonamide antibiotic residues in the different fish species varied widely among the cities, and the highest level of antibiotic residues was found in the muscle of carnivorous fish. The results from a health risk evaluation on the consumption of these fish indicated that the risk from long-term antibiotic exposure to local residents from the intake of the sampled fish was small and not sufficient to pose a significant health risk to consumers.

## 1. Introduction

Fish are rich in nutrients and provide one of the most important sources of nutrition in many human societies [1]. In the past few decades, aquacultural production of fish for human consumption has flourished worldwide. In China, freshwater fish farming is conducted through various methods, such as pond farming, lake farming, reservoir farming, and river farming. However, pond farming is the most commonly used method, accounting for the largest share in terms of both farming area and fish production. In many regions, the use of certain antibiotics is permitted in aquaculture. Moderate use of antibiotics in intensive large-scale fish farming can help prevent and control diseases in fish that occur in crowded environments [2,3], thereby reducing fish mortality and minimizing production losses for fish farmers. This ensures that producers can obtain stable incomes and generate economic benefits. Additionally, antibiotics may also have varying degrees of growth-promoting effects on animals, which means that farmers can expedite the time it takes to bring fish to the market and generate sales revenue in advance, thereby enhancing economic efficiency. However, in these fish farming practices, improper use of antibiotics can lead to the excessive presence of antibiotics in the environment, resulting in the accumulation of antibiotics in water bodies. Different concentrations of antibiotics, including sulfonamide antibiotics such as sulfamethoxazole, sulfamethazine, sulfadiazine, sulfathiazole, and trimethoprim, have been detected in water bodies near large-scale livestock and poultry farms in Jiangsu [4] and Zhejiang [5] provinces in China. On the other hand, antibiotics may also persist in fish products, entering the human body through the food chain, leading to the accumulation of antibiotics in the human body [6].

The accumulation of antibiotics in the human body can disrupt the equilibrium between beneficial and pathogenic bacteria in the intestinal tract, resulting in disturbances in the gut microbiota and consequent intestinal issues like diarrhea and gastrointestinal discomfort [7]. Additionally, a more serious consequence is the potential development of antibiotic-resistant bacteria due to the long-term accumulation of antibiotics [7]. This, in turn, leads to more difficult treatment of bacterial infections, with limited treatment options, increased costs, and potentially more toxic antibiotic therapies [8]. The health risks associated with antibiotic accumulation have raised concerns worldwide. Many governments, such as those of the United States, the European Union, and China, have successively established strict regulations and measures to control antibiotic residues in fish products [9,10,11]. However, there are challenges and issues associated with monitoring and regulating antibiotics in fish products. Antibiotics encompass various types, and different categories may require distinct analytical methods for detection, thereby complicating monitoring efforts. Additionally, antibiotic residues in fish are typically found at very low levels, often in the ng/g range or even lower [7]. Consequently, highly sensitive analytical methods are necessary to accurately detect antibiotic residues, creating an additional challenge for laboratory personnel. In response to these difficulties and challenges, close collaboration between regulatory agencies, research institutions, and industry experts is crucial. This collaboration should involve active engagement in research and technological innovation. It should encompass continuous improvement of analytical methods, the establishment of standards and guidelines, the promotion of technological advancements, and the reinforcement of regulatory and educational measures to ensure control and safety regarding antibiotic residues in food. Simultaneously, alongside strengthening the regulation of antibiotics in fish products, initiatives should actively pursue alternative methods to reduce antibiotic dependency in aquaculture. These efforts involve optimizing farming environments, improving water quality management, maintaining appropriate stocking densities, optimizing feed formulations, and selectively breeding superior fish fry. The objective is to enhance fish immune systems, reduce the risk of disease outbreaks, and ultimately minimize antibiotic use.

In order to verify compliance with and the effectiveness of regulations, it is necessary to routinely test antibiotic residues in fish sold for human consumption and evaluate their associated health risks. Currently, the main types of antibiotics found in water environments, as well as in related aquatic products, include macrolides, β-lactams, sulfonamides, tetracyclines, aminoglycosides, and quinolones [12]. Among these, sulfonamide antibiotics is the class of antibiotics that are detected most frequently and in higher amounts [13,14]. Sulfonamides have broad-spectrum antibacterial properties and are convenient to use at a low price, making them widely used in livestock farming and aquaculture. However, experimental evidence has shown that sulfonamides may have an impact on the metabolism in fish, resulting in significant deformities and lethal effects when fish are cultured in a laboratory using sulfonamides [15]. Additional studies have demonstrated that nearly all sulfonamides exhibit toxic effects on algae (EC_50_: 1.54–32.25 mg/L), with sulfamethoxazole (EC_50_ = 6.2 μmol/L), sulfadiazine (EC_50_ = 4.9 μmol/L), and sulfamethoxypyridazine (EC_50_ = 13.64 μmol/L) being among the most toxic [16]. 

Sulfonamides have been detected to varying degrees in Chinese freshwater bodies, and in aquatic food products extracted from these water bodies, for example in Taihu Lake, Dongting Lake, and the Xiangjiang River [17,18,19], mainly including sulfamethoxazole, sulfadiazine, sulfadoxine, and sulfapyridine. At present, few studies have been conducted on antibiotic residues in freshwater fish from northeastern China. Therefore, it is necessary to detect antibiotic residues in major edible fish species in this region and assess their health risks. In the present study, a total of 10 commercially available fish species were purchased in Shenyang, Changchun, and Harbin, respectively, and the levels of 12 sulfonamide antibiotics in edible fish were examined and their health risks were evaluated.

## 2. Materials and Methods

### 2.1. Study Area and Sample Collection

**Figure 1:**
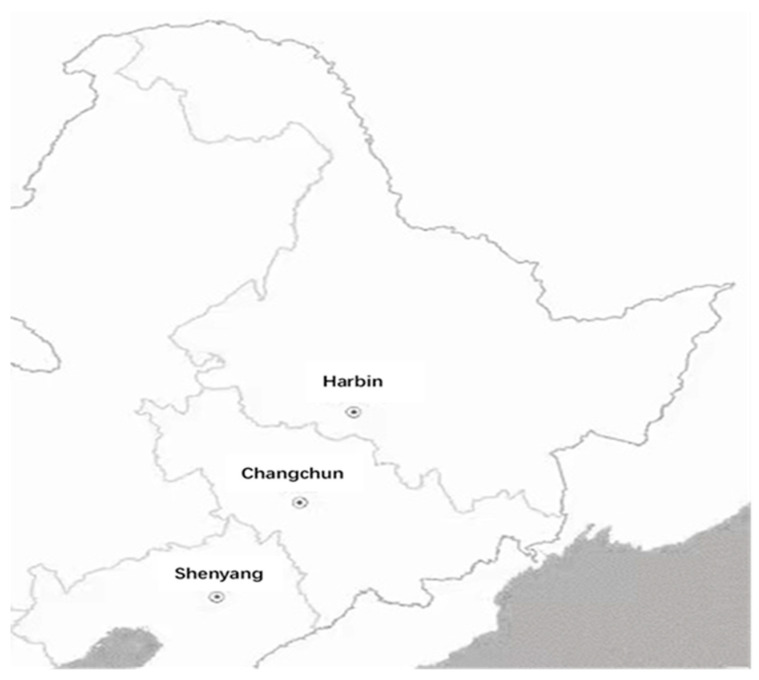
The map of sampling points.

In this study, 223 fish samples from 10 species were collected from local markets in three areas (Harbin, Changchun, and Shenyang) (Figure 1). All the fish purchased were frozen and deceased. The collected fish species included *Siniperca chuatsi*, *Aristichthys nobilis*, *Carassius auratus*, *Cyprinus carpio*, *Phoxinus lagowskii*, *Hemisalanx prognathus*, *Ctenopharyngodon idellus*, *Silurus asotus*, *Hypophthalmichthys molitrix*, and *Pelteobagrus fulvidraco*. The details of the fish sample information are given in Table 1. The fish were stored in ice boxes and transported to the laboratory immediately. As the local population consumed fish muscle most often, only fish muscle was examined for its antibiotic content in this study. The fish muscles were homogenized, weighed, and frozen at −20 °C until analysis.

### 2.2. Sample Preparation and Instrumental Analysis

In the present study, 12 sulfonamides (sulfapyridine, SPD; sulfadiazine, SDZ; sulfacetamide, SA; sulfamethoxazole, SMX; sulfachloropyridazine, SCP; sulfathiazole, STZ; sulfisoxazole, SIA; sulfamethazine, SMT; sulfamerazine, SMR; sulfamonomethoxine, SMM; sulfamethoxypyridazine, SMP; sulfadimethoxine, SDM) were analyzed in the muscles of 10 fish species.

Sample treatment followed the methods from previously published studies [20,21], but with some improvements. Briefly, approximately 5 g wet weight (ww) of fish muscle sample was spiked with two surrogate standards (sulfamethoxazole -D_4_ and sulfathiazole -D_4_). Ethyl acetate (20 mL) was added for analytes extraction, and the sample was then vortexed followed by sonication for 20 min. After centrifugation at 6000 rpm for 5 min, the supernatant was transferred into a sterile pear-shaped flask. The extraction procedure was repeated two times, and the obtained supernatants were combined. Then, hydrochloric acid (0.1 mol/L, 4 mL) was added into the combined supernatants for protein precipitation and the sample was then evaporated to nearly 3 mL by rotary evaporation. 

The supernatant was transferred to a new polytetrafluoroethylene centrifuge tube and 3 mL n-hexane was added. After centrifugation at 3000 rpm for 5 min, the n-hexane layer was removed. The extraction procedure was repeated two times, and the obtained extracts were combined and submitted to a clean-up step using an SPE MCX cartridge (3 mL, 60 mg; Waters, Massachusetts, USA), which was preconditioned with 2 mL of methanol and 2 mL 0.1 mol/L of HCl. Then, the extract was loaded into the cartridge. Each cartridge was rinsed with 1 mL 0.1 mol/L of HCl and 2 mL of methanol/acetonitrile mixture (1:1, *v*/*v*) and dried with a gentle stream of nitrogen. Using 6 mL of 5% ammonium methanol eluted, the eluate was evaporated to near dryness and, lastly, the residue was reconstituted with 10% methanol in water.

The identification of 12 antibiotics was performed using an AB Sciex high-performance liquid chromatography (UPLC) system coupled, with a 3200 QTRAP electrospray triple quadrupole mass spectrometer (ESI–MS/MS). For the separation of pharmaceuticals, an Agilent Infinity Lab Poroshell 120 EC-C18 column (100 mm × 2.1 mm, 2.7 µm; Agilent, USA) was used. Quantitative analysis of the target compounds was operated in multiple reaction monitoring (MRM) mode, using an electrospray ionization source operating in positive mode. More details about the instrumental analysis are described in the Supplementary Material and Table S1.

### 2.3. Quality Assurance and Quality Control

In this study, the procedural blanks were performed after every five samples. Spiking experiments on the antibiotics (*n* = 3) were performed in *Aristichthys nobilis*. The average recovery rate for antibiotics was 67–117% (Table 2). The method quantification limits (MQLs) for the target antibiotics were calculated based on 10 times the S/N values, which ranged from 0.006~1.0 ng/g ww (Table 2). For the samples with target concentrations below the MQLs, a value of 0 was assigned for the calculations.

### 2.4. Human Health Risk Assessment

The estimated daily intake (EDI), hazard quotient (HQ), and total hazard quotient (THQ) were calculated based on the antibiotics concentrations in the fish samples, which were used for the human health risk assessment for the local people who consumed the fish.

The EDI was computed as Equation (1):

(1) $$\mathrm{EDI}=\frac{C\times \mathrm{IR}}{\mathrm{BW}}$$

where EDI is the daily intake rate of the antibiotics (μg/kg bw/d) and C is the measured antibiotics concentration in fish (ng/g ww). In this study, the consumer’s weight (BW) and the fish ingestion rate of the consumer (IR) for different age groups were derived from a previous study and recent surveys [22,23], (see Supplementary Material for details).

The HQ indicates non-carcinogenic risks, which were calculated according to Equation (2) [24]:

(2) $$\mathrm{HQ}=\frac{\mathrm{EDI}}{\mathrm{ADI}}$$

where ADI is the acceptable daily intake of an antibiotic (μg/kg bw/d) [25].

Another index, the THQ, is also often calculated to assess the risks from coexisting pollutants. This index was computed by summing up the HQ of 12 sulfonamides:

(3) $$\mathrm{THQs}\left(sulfonamides\right)=\mathrm{HQ}\left(sulfapyridine\right)+\mathrm{HQ}\left(sulfacetamide\right)+\mathrm{HQ}(sulfamethoxazole)$$

## 3. Results

### 3.1. Antibiotic Residues

The antibiotic residues from fish samples from the markets in Changchun, Shenyang, and Harbin are shown in Table 3. All twelve sulfonamide antibiotics were detected in the fish to different degrees. The total detection rates among all the fish samples in the three cities were: SMX (93.48%) > SMT (84.78%) > SIA (82.61%) > SCP (80.43%) > SDM (65.22%) > STZ (63.04%) > SA (48.91%) > SMR (45.65%) > SMM (41.30%) > SPD (39.13%) = SMP (39.13%) > SDZ (36.96%). Among the nine fish species sold in Harbin, the highest total detection rate was for SMX and was 87.88%, and the lowest detection rate was for SMP and was 30.30%. Among the eight fish species sold in Changchun, the highest total detection rate was for SMX and was 92.86%, and the lowest detection rate was for SPD and was 25.00%. Among the nine fish species sold in Shenyang, the highest detection rate was for SMX and was 100%, and the lowest detection rate was for SDZ and was 35.48%. The data showed that the detection rates for SMX were the highest in all three cities, and were greater than 85%, while SPD, SDZ, and SMP were the three antibiotics with the lowest detection rates, with detection rates of less than 40%. 

It is worth noting that all the fish samples, not only had the highest detection rates and the highest average contents of SMX, but the average concentration of SMX in fish muscle in Harbin, Changchun, and Shenyang was 4.23 ng/g ww, 2.85 ng/g ww, and 5.22 ng/g ww, respectively. The highest SMX concentration found was 5.28 ng/g ww in the muscle of Changchun *Hypophthalmichthys molitrix*, 8.72 ng/g ww in the muscle of Shenyang *Phoxinus lagowskii*, and 9.49 ng/g ww in the muscle of Harbin *Silurus asotus* (Tables S2–S4). 

**Figure 2:**
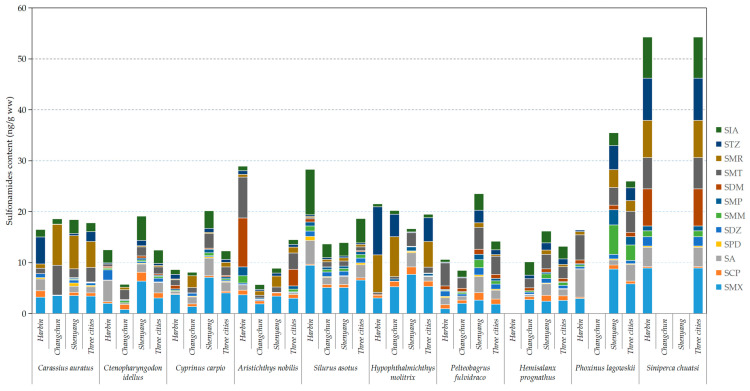
The content of sulfonamides in various fish species from Harbin, Changchun, Shenyang, and the total for the three cities.

The status of antibiotic residues in fish is closely related to the living habits, nutritional level, and natural environment of the fish. In this study, the residues in fish samples from the three cities were slightly different, and the fish with the highest 12 sulfonamide antibiotic residues in commercially available fish from Changchun, Shenyang, and Harbin were *Hypophthalmichthys molitrix*, *Phoxinus lagowskii*, and *Siniperca chuatsi*, respectively (Figure 2). A summary analysis of the samples from all three cities revealed that the total antibiotic concentrations across the different fish species were in the order: *Siniperca chuatsi* > *Phoxinus lagowskii* > *Hypophthalmichthys molitrix* > *Silurus asotus* > *Carassius auratus* > *Aristichthys nobilis* > *Pelteobagrus fulvidraco* > *Hemisalanx prognathus* > *Ctenopharyngodon idellus* > *Cyprinus carpio* (Figure 2).

### 3.2. Dietary Risk Assessment of Sulfonamide Antibiotics in Fish

The estimated daily intake (EDI) values of residents in the three cities for the 12 sulfonamide antibiotics, through the consumption of the 10 sampled fish species, are given in Table 4. The results show that the daily intake of Σ12 sulfonamide antibiotics through fish consumption was 0.019–0.095, 0.008–0.050, and 0.004–0.027 μg/kg bw/day for residents of Harbin in three age groups (2–5 years, 6–17 years, and ≥18 years), respectively. The daily intake of Σ12 sulfonamide antibiotics through fish consumption in Changchun city was 0.010–0.035, 0.005–0.019, and 0.003–0.010 μg/kg bw/day, respectively. The daily intake of sulfonamide antibiotics through fish consumption in Shenyang city was 0.032–0.062, 0.008–0.033, and 0.008–0.012 μg/kg bw/day, respectively. Table 5 presents the target hazard quotients (THQs) for the sulfonamides in children, youths, and adults. It is evident that all the observed THQ values were below 1.

## 4. Discussion

In this study, we showed that SMX was the most prevalent sulfonamide antibiotic in all fish species investigated, and also that all the sulfonamide antibiotics investigated could be found in fish muscle. This is important because fish play a crucial role in the human diet as a valuable source of protein. Accordingly, the assessment of fish consumption safety holds significant importance. However, the escalating problem of water pollution due to rapid urbanization poses a substantial threat to the safety of fish [26]. Furthermore, China stands as a prominent nation in terms of antibiotic use and production, thereby raising concerns about the presence of antibiotic residues in the environment. Previous studies examining water and fish samples from other regions of China have also identified SMX as the predominant sulfonamide antibiotic present in environmental samples [27,28,29]. Zhao et al. collected fish samples from water bodies near the Pearl River Delta and examined the levels of 26 types of antibiotics in fish, and among the nine sulfonamide antibiotics they targeted, SMX was found to have the highest detection rate of 68%, with the highest concentration reaching 8 ng/g ww [29]. Chen et al. [27] studied sulfonamide contamination in marine products and sea water. They reported that 3 ng/g of SMX was found in the sampled marine products (shrimps, crabs, mollusks, and fish), which is similar to the results from this study. 

SMX is one of the sulfonamide antibiotics currently permitted for use in aquaculture in China [2], and the scale of production and use of SMX in China is at a high level compared to global levels [12]. This may be one of the reasons for the high concentrations of SMX residues in the fish sampled in this study. In addition to SMX, SDZ and SMT are also currently permitted antibiotics in China, therefore, in this experiment, the average level of SMT detected in fish muscle was also high, but the amount of SDZ detected was relatively low. Previous laboratory simulation experimental studies have shown that SDZ has a relatively low Kow value [30], as well as a low BCF value [18,31], compared to other sulfonamide antibiotics. These factors are not conducive to its persistent accumulation in the organism, and the low SDZ content in the fish in this study may be due to these effects.

The lowest levels of sulfonamides detected in the three northeastern cities were all SPD, which is not allowed to be used as a veterinary drug in China and its use is gradually being stopped in China because of its strong side effects [2]. The small amount of SPD accumulation in the fish samples is likely to come from enrichment in the natural environment, suggesting that accumulation in the environment still exists despite the gradual discontinuation of the production and use of this drug, hence such legacy environmental accumulation of antibiotics deserves attention in the future.

The average levels of sulfonamide antibiotics detected in fish from Harbin, Changchun, and Shenyang were 1.83 ng/g ww, 0.98 ng/g ww, and 1.60 ng/g ww, respectively. Liu et al. [2] examined reported sulfonamide antibiotic residues in fish from the Chinese aquaculture industry and found that they varied widely, ranging from 0.01–100 ng/g ww, with an average concentration of 61.1 ng/g ww. Compared with these results, the sulfonamide antibiotic residues in fish from the three northeastern cities in this study were at low-to-medium levels. Sulfonamide antibiotics are likewise, a commonly detected antibiotic in fishery products from Thailand [32], Argentina [33], Iran [34], and Brazil [35], and Chile [36]. It is worth noting that although the current data suggest that the concentration levels of sulfonamide antibiotics in fish are mostly within safe limits, the presence of sulfonamide-resistant genes has been detected in fish from different regions. A study from Ho Chi Minh City in Vietnam found that, in comparison to fresh seafood, 32.56%, 40%, and 10% of *Salmonella* showed resistance to sulfamethoxazole in fish, squid, and shrimp, respectively [37]. Additionally, the study identified the presence of sulfonamide resistance genes, *sul 1* and *sul 2*. Another study, from India, also identified that 21% of fish *Escherichia coli* carried multidrug-resistant (MDR) genes, including the sulfonamide resistance gene *sul 1* [38]. Therefore, the residues of sulfonamide antibiotics in aquatic products deserves more attention, and more extensive testing should be conducted to explore their residues and provide data to support the evaluation of the health risks from their consumption.

In this study, carnivorous fish (*Siniperca chuatsi*, *Silurus asotus*), omnivorous fish (*Pelteobagrus fulvidraco*, *Aristichthys nobilis*, *Hypophthalmichthys molitrix*; *Carassius auratus*, *Cyprinus carpio*; *Phoxinus lagowskii*, *Hemisalanx prognathus*), and herbivorous fish (*Ctenopharyngodon idellus*) had mean mass concentrations of sulfonamide antibiotics in their bodies of 2.31 ng/g ww, 1.34 ng/g ww, and 1.04 ng/g ww, respectively. Hence, the average concentration of antibiotic residues were in the order carnivorous fish > omnivorous fish > herbivorous fish, which is consistent with the results by Liu et al. [18] and Ye et al. [28]. A study conducted on the levels of sulfonamide antibiotics in the food web of Laizhou Bay, China, revealed that sulfonamides undergo bioaccumulation and biomagnification in the marine food chain [39]. This could be attributed to the fact that sulfonamides, such as sulfamethoxazole, are well absorbed in the intestinal tracts, but not efficiently metabolized in aquatic organisms like crustaceans, mollusks, and fish. As a result, the trophic magnification of sulfonamides in the food chain is likely due to their limited metabolic transformation and efficient assimilation in animals at higher trophic levels. However, the samples in our study were mostly omnivorous fish, and the sample sizes of carnivorous and herbivorous fish were small, so there may be a bias in the statistical results, and a future study of antibiotic enrichment patterns in fish may need to expand the sample size or conduct laboratory simulation experiments.

In comparison with previous studies, the daily intake of antibiotics from fish consumption in Changchun and Shenyang for all age groups in this study was found to be lower than the EDI value for Yellow Sea fish (0.065 μg/kg bw/day) [40], although the EDI value for antibiotics from *Siniperca chuatsi* consumption in Harbin for age groups 2–5 was slightly higher at 0.095 μg/kg bw/day. It should be noted that the EDI values clearly showed a trend of higher values with lower age in the three groups. It suggests a higher health risk among younger age groups, when fish containing antibiotics are consumed across different populations. In China, fish is a primary source of high-quality protein for the population. However, the presence of residual antibiotics in fish may pose a health risk to consumers, particularly children and adolescents. The underdeveloped intestinal and hepatic systems in children result in a reduced capacity to metabolize antibiotics compared to adults [41]. Furthermore, their immature immune systems make them more susceptible to antibiotic-resistant bacteria [42]. Studies conducted on mice have demonstrated that exposure to low concentrations of residual antibiotics during developmental stages can disrupt metabolism, gut microbiota, and adipogenesis, potentially increasing the risk of obesity and diabetes [43,44]. Therefore, special attention should be paid to safety regarding fish consumed by children.

In this study, the target hazard quotients (THQs) for sulfonamides were also estimated for children, adolescents, and adults. The results revealed that the THQ values for all three age groups were below 1. This indicates that the daily intake of the twelve sulfonamides did not exceed the reference doses. Therefore, the health risk associated with the consumption of the ten fish species that were studied is considered to be low for the local population.

In general, the levels of antibiotics in fish in this study were below the prescribed limit and the health risks were low. However, trace antibiotic residues in fish may still exert selective pressure on bacteria and induce the formation of antibiotic resistant bacteria (ARB) and antibiotic resistance genes (ARGs). ARGs may accumulate in humans through the transfer of aquatic products, posing a potential threat to human health in the long term. Therefore, the problem of trace antibiotic residues in aquatic products still deserves discussion and attention.

To mitigate antibiotic residues in fish, the following strategies are recommended: (1) The aquaculture industry should employ antibiotics judiciously, limiting their use to necessary cases and adhering to prescribed safe dosage guidelines. (2) The government should enhance regulation and oversight of the aquaculture industry and food production processes to ensure appropriate antibiotic use and prevent misuse. Additionally, regular monitoring of antibiotic residues in fish is necessary. (3) Improvements in aquaculture management practices are necessary, including measures such as water quality management, the control of stocking density, and disease prevention and control, to reduce the dependence on antibiotics. (4) Encouraging the use of safer and environmentally friendly alternatives to antibiotics in fish farming should be a priority. (5) Increasing public awareness and education campaigns are crucial to foster a better understanding of the hazards associated with antibiotic misuse among the general public and stakeholders in the aquaculture industry.

## 5. Conclusions

In this study, 10 commercially available freshwater fish species were collected from the cities of Changchun, Shenyang, and Harbin in northeastern China, and the fugitive status of sulfonamide antibiotics in the fish was examined. The results showed that the average levels of sulfonamide antibiotics from Harbin, Changchun, and Shenyang were 1.83 ng/g ww, 0.98 ng/g ww, and 1.60 ng/g ww, respectively. The highest concentrations found in all the fish samples were of SMX, with average concentrations of 4.23 ng/g ww, 2.85 ng/g ww, and 5.22 ng/g ww from Harbin, Changchun, and Shenyang, respectively. The highest detection rates for SMX were greater than 85%. The lowest mean concentrations in all the fish samples were of SPD and were 0.19 ng/g ww, 0.06 ng/g ww, and 0.24 ng/g ww, in the three cities, respectively. The content of sulfonamide antibiotics in the different fish species varied widely among the cities and, overall, the highest antibiotic content was in the muscle of carnivorous fish. The results of a health risk evaluation on the consumption of these fish indicated that the risk from long-term antibiotic exposure to local residents from the intake of the sampled fish was small and not sufficient to pose a significant health risk to consumers.

## Figures and Tables

**Table 1:** Sample information on each fish species.

City	Feeding Habits	Species	Sample Size	Length (cm)	Weight (g)
**Harbin**	***Carnivorous***	*Silurus asotus*	4	42~48	1000
		*Siniperca chuatsi*	4	33~35	750
	***Omnivorous***	*Cyprinus carpio*	4	33~36	1000
		*Carassius auratus*	4	22~28	500
		*Phoxinus lagowskii*	24	12~18	17
		*Aristichthys nobilis*	3	52~55	2000
		*Pelteobagrus fulvidraco*	6	18~20	100
		*Hypophthalmichthys molitrix*	4	45~50	1900
	***Herbivorous***	*Ctenopharyngodon idellus*	4	41~44	1250
**Shenyang**	***Carnivorous***	*Silurus asotus*	4	40~45	650
	***Omnivorous***	*Hemisalanx prognathus*	40	7~9	5
		*Cyprinus carpio*	4	36~40	900
		*Carassius auratus*	4	26~32	550
		*Phoxinus lagowskii*	30	12~18	16
		*Aristichthys nobilis*	3	53~55	2000
		*Pelteobagrus fulvidraco*	6	23~26	250
		*Hypophthalmichthys molitrix*	3	51~55	1800
	***Herbivorous***	*Ctenopharyngodon idellus*	4	41~43	1200
**Changchun**	***Carnivorous***	*Silurus asotus*	4	39~43	600
	***Omnivorous***	*Hemisalanx prognathus*	40	7~9	3
		*Cyprinus carpio*	4	38~39	900
		*Carassius auratus*	4	26~28	400
		*Aristichthys nobilis*	3	52~57	2000
		*Pelteobagrus fulvidraco*	6	21~24	200
		*Hypophthalmichthys molitrix*	3	52~55	2000
	***Herbivorous***	*Ctenopharyngodon idellus*	4	36~42	800

**Table 2:** Recovery rate, method detection limits, and method quantification limits.

Compounds	Surrogate	Recovery %	MDLs ng/g ww	MQLs ng/g ww
sulfapyridine	sulfamethoxazole -D_4_	79 ± 8	0.04	0.12
sulfadimethoxine	sulfathiazole -D_4_	117 ± 7	0.002	0.006
sulfamethoxazole	sulfamethoxazole -D_4_	90 ± 5	0.002	0.006
sulfachloropyridazine	sulfamethoxazole -D_4_	88 ± 7	0.004	0.01
sulfathiazole	sulfathiazole -D_4_	85 ± 6	0.02	0.06
sulfisoxazole	sulfathiazole -D_4_	90 ± 8	0.06	0.20
sulfadiazine	sulfamethoxazole -D_4_	96 ± 8	0.20	0.50
sulfamethazine	sulfathiazole -D_4_	87 ± 3	0.02	0.06
sulfamerazine	sulfathiazole -D_4_	67 ± 7	0.20	0.60
sulfamonomethoxine	sulfathiazole -D_4_	112 ± 4	0.06	0.20
sulfamethoxypyridazine	sulfathiazole -D_4_	110 ± 8	0.06	0.20
sulfacetamide	sulfamethoxazole -D_4_	70 ± 5	0.40	1.00

**Table 3:** Summary of the results on the sulfonamides detection frequencies (percentage of all samples) and concentrations (ng/g ww) in fish samples.

s	Harbin		Changchun		Shenyang		Summary of Three Cities	
	Concentration (Mean)	Frequency %	Concentration (Mean)	Frequency %	Concentration (Mean)	Frequency %	Concentration (Mean)	Frequency %
SMX	4.23	87.88	2.85	92.86	5.22	100	4.15	93.48
SCP	0.52	78.79	0.63	78.57	0.98	83.87	0.71	80.43
SA	2.60	57.58	0.52	28.57	1.90	58.06	1.72	48.91
SPD	0.19	42.42	0.06	25.00	0.24	48.39	0.17	39.13
SDZ	0.84	45.45	0.32	28.57	0.57	35.48	0.58	36.96
SMM	0.50	33.33	0.21	32.14	1.11	58.06	0.62	41.30
SMP	0.47	30.30	0.19	28.57	0.77	58.06	0.49	39.13
SDM	2.24	84.85	0.16	39.29	0.38	67.74	0.95	65.22
SMT	2.97	84.85	1.82	78.57	2.42	90.32	2.43	84.78
SMR	1.89	39.39	2.49	50.00	1.66	48.39	2.00	45.65
STZ	2.85	72.73	0.76	42.86	1.34	70.97	1.68	63.04
SIA	2.68	78.79	1.32	78.57	2.59	90.32	2.23	82.61

**Table 4:** Estimated daily intake (EDI) of Σ12 sulfonamides (μg/kg bw/day) via the consumption of ten species of fish in three cities.

Species	Harbin			Changchun			Shenyang		
	Age: 2–5	Age: 6–17	Age: ≥18	Age: 2–5	Age: 6–17	Age: ≥18	Age: 2–5	Age: 6–17	Age: ≥18
*Carassius auratus*	0.029	0.015	0.008	0.032	0.017	0.009	0.032	0.017	0.009
*Ctenopharyngodon idellus*	0.022	0.012	0.006	0.010	0.005	0.003	0.033	0.018	0.009
*Cyprinus carpio*	0.015	0.008	0.004	0.014	0.007	0.004	0.035	0.019	0.010
*Aristichthys nobilis*	0.051	0.027	0.014	0.010	0.005	0.003	0.016	0.008	0.004
*Silurus asotus*	0.049	0.026	0.014	0.024	0.013	0.007	0.024	0.013	0.007
*Hypophthalmichthys molitrix*	0.038	0.020	0.011	0.035	0.019	0.010	0.029	0.015	0.008
*Pelteobagrus fulvidraco*	0.019	0.010	0.005	0.015	0.008	0.004	0.041	0.022	0.012
*Hemisalanx prognathus*	-	-	-	0.018	0.009	0.005	0.028	0.015	0.008
*Phoxinus lagowskii*	0.029	0.015	0.008	-	-	-	0.062	0.033	0.018
*Siniperca chuatsi*	0.095	0.050	0.027	-	-	-	-	-	-

**Table 5:** Total hazard quotients (THQs) for Σ12 sulfonamides from the consumption of ten species of fish in three cities.

Species	Harbin			Changchun			Shenyang		
	Age: 2–5	Age: 6–17	Age: ≥18	Age: 2–5	Age: 6–17	Age: ≥18	Age: 2–5	Age: 6–17	Age: ≥18
*Carassius auratus*	0.0006	0.0003	0.0002	0.0006	0.0003	0.0002	0.0006	0.0003	0.0002
*Ctenopharyngodon idellus*	0.0004	0.0002	0.0001	0.0002	0.0001	0.0001	0.0007	0.0004	0.0002
*Cyprinus carpio*	0.0003	0.0002	0.0001	0.0003	0.0001	0.0001	0.0007	0.0004	0.0002
*Aristichthys nobilis*	0.0010	0.0005	0.0003	0.0002	0.0001	0.0001	0.0003	0.0002	0.0001
*Silurus asotus*	0.0010	0.0005	0.0003	0.0005	0.0003	0.0001	0.0005	0.0003	0.0001
*Hypophthalmichthys molitrix*	0.0008	0.0004	0.0002	0.0007	0.0004	0.0002	0.0006	0.0003	0.0002
*Pelteobagrus fulvidraco*	0.0004	0.0002	0.0001	0.0003	0.0002	0.0001	0.0008	0.0004	0.0002
*Hemisalanx prognathus*	-	-	-	0.0004	0.0002	0.0001	0.0006	0.0003	0.0002
*Phoxinus lagowskii*	0.0006	0.0003	0.0002	-	-	-	0.0012	0.0007	0.0004
*Siniperca chuatsi*	0.0019	0.0010	0.0005	-	-	-	-	-	-

## Data Availability

Not applicable.

## Supplementary Materials

The following supporting information can be downloaded at: https://www.mdpi.com/article/10.3390/toxics11100835/s1, Table S1: Optimized UPLC–MS/MS parameters; Table S2: Antibiotic Content in Harbin Fish (ng/g ww); Table S3: Antibiotic Content in Changchun Fish (ng/g ww); Table S4: Antibiotic Content in Shenyang Fish (ng/g ww).

## References

[1] Guérin T., Chekri R., Vaste C., Sirot V., Volatier J.L., Leblanc J.C., Noël L. (2011). Determination of 20 trace elements in fish and other seafood from the french market. Food Chem..

[2] Liu X., Steele J.C., Meng X.Z. (2017). Usage, residue, and human health risk of antibiotics in Chinese aquaculture: A review. Environ. Pollut..

[3] Zhang Q.Q., Ying G.G., Pan C.G., Liu Y.S., Zhao J.L. (2015). Comprehensive evaluation of antibiotics emission and fate in the river basins of China: Source analysis, multimedia modeling, and linkage to bacterial resistance. Environ. Sci. Technol..

[4] Wei R., Ge F., Huang S., Chen M., Wang R. (2011). Occurrence of veterinary antibiotics in animal wastewater and surface water around farms in Jiangsu Province, China. Chemosphere.

[5] Wang J., Wu H., Qian M., Ma J., Zhang H., Yang H. (2018). Occurrence and Distribution of Veterinary Residues in Aquatic Environments near Livestock and Poultry Farms in Zhejiang Province, China. Asian J. Chem..

[6] WHO (2014). Antimicrobial resistance: Global report on surveillance. Australas. Med. J..

[7] Li S., Shi W., Liu W., Li H., Zhang W., Hu J., Ke Y., Sun W., Ni J. (2018). A duodecennial national synthesis of antibiotics in China’s major rivers and seas (2005–2016). Sci. Total. Environ..

[8] Xiong W., Sun Y., Zhang T., Ding X., Li Y., Wang M., Zeng Z. (2015). Antibiotics, antibiotic resistance genes, and bacterial community composition in fresh water aquaculture environment in China. Microb. Ecol..

[9] Centner T.J. (2016). Recent government regulations in the United States seek to ensure the effectiveness of antibiotics by limiting their agricultural use. Environ. Int..

[10] Hvistendahl M. (2012). China takes aim at rampant antibiotic resistance. Science.

[11] Ruiz-Viceo J.A., Rosales-Conrado N., Guillén-Casla V., Pérez-Arribas L.V., León-González M.E., Polo-Díez L.M. (2012). Fluoroquinolone antibiotic determination in bovine milk using capillary liquid chromatography with diode array and mass spectrometry detection. J. Food. Compos. Anal..

[12] Lulijwa R., Rupia E.J., Alfaro A.C. (2020). Antibiotic use in aquaculture, policies and regulation, health and environmental risks: A review of the top 15 major producers. Rev. Aquacult..

[13] Gray A.D., Todd D., Hershey A.E. (2020). The seasonal distribution and concentration of antibiotics in rural streams and drinking wells in the piedmont of North Carolina. Sci. Total Environ..

[14] Hirsch R., Ternes T., Haberer K., Kratz K.L. (1999). Occurrence of antibiotics in the aquatic environment. Sci. Total. Environ..

[15] Ska A.B., Stolte S., Arning J., Uebers U., Boschen A., Stepnowski P., Matzke M. (2011). Ecotoxicity evaluation of selected sulfonamides. Chemosphere.

[16] Baran W., Sochacka J., Wardas W. (2006). Toxicity and biodegradability of sulfonamides and products of their photocatalytic degradation in aqueous solutions. Chemosphere.

[17] Chen L., Li H., Liu Y., Cui Y., Li Y., Yang Z. (2020). Distribution, residue level, sources, and phase partition of antibiotics in surface sediments from the inland river: A case study of the Xiangjiang River, south-central China. Environ. Sci. Pollut. Res..

[18] Liu X., Lu S., Meng W., Zheng B. (2018). Residues and health risk assessment of typical antibiotics in aquatic products from the Dongting Lake, China-“Did you eat “antibiotics” today?. Environ. Sci. Pollut. Res..

[19] Zhou L.J., Wang W.X., Lv Y.J., Mao Z.G., Chen C., Wu Q.L. (2020). Tissue concentrations, trophic transfer and human risks of antibiotics in freshwater food web in Lake Taihu, China. Ecotox. Environ. Safe..

[20] Chiesa L.M., Nobile M., Panseri S., Arioli F. (2017). Antibiotic use in heavy pigs: Comparison between urine and muscle samples from food chain animals analysed by HPLC-MS/MS. Food Chem..

[21] (2013). Determination of Sulfonamides residues in animal derived food by High Performance Liquid Chromatographic method.

[22] Ministry of Agriculture the PRC (Ministry of Agriculture of the People’s Republic of China) (2002). Bulletin (NO. 235): The Guideline of Maximum Residue Limits for Veterinary Drugs in Animal Food.

[23] Shen M., Kang C., Song T., Lu H., Wang Y., Yu B., Wang R., Cheng J. (2020). Content and health risk assessment of heavy metals and polybrominated diphenyl ethers in fish from Songhua Lake (Jilin City), China. Environ. Sci. Pollut. Res..

[24] USEPA (2011). Regional Screening Level (RSL) Summary Table 2011.

[25] (2019). National Food Safety Standard-Maximum Residue Limits for Veterinary Drugs in Foods.

[26] Li C., Zhang C., Zhong S., Duan J., Li M., Shi Y. (2023). The Removal of Pollutants from Wastewater Using Magnetic Biochar: A Scientometric and Visualization Analysis. Molecules.

[27] Chen H., Shan L., Xu X.R., Liu S.S., Zhou G.J., Sun K.F., Zhao J.L., Ying G.G. (2015). Antibiotics in typical marine aquaculture farms surrounding hailing island, South China: Occurrence, bioaccumulation and human dietary exposure. Mar. Pollut. Bull..

[28] Ye C., Shi J., Zhang X., Qin L., Jiang Z., Wang J., Li Y., Liu B. (2021). Occurrence and bioaccumulation of sulfonamide antibiotics in different fish species from Hangbu-Fengle River, Southeast China. Environ. Sci. Pollut. Res..

[29] Zhao J.L., Liu Y.S., Liu W.R., Jiang Y.X., Su H.C., Zhang Q.Q., Chen X.W., Yang Y.Y., Chen J., Liu S.S. (2015). Tissue-specific bioaccumulation of human and veterinary antibiotics in bile, plasma, liver and muscle tissues of wild fish from a highly urbanized region. Environ. Pollut..

[30] Li N., Zhao X., Wu W., Zhao X. (2014). Occurrence, seasonal variation and risk assessment of antibiotics in the reservoirs in North China. Chemosphere.

[31] Xie Z., Tang J., Wu X., Fan S., Cheng H., Li X., Hua R. (2019). Bioconcentration and ecotoxicity of sulfadiazine in the aquatic midge *Chironomus riparius*. Environ. Toxicol. Phar..

[32] Jansomboon W., Boontanon S.K., Boontanon N., Polprasert C., Da C.T. (2016). Monitoring and determination of sulfonamide antibiotics (sulfamethoxydiazine, sulfamethazine, sulfamethoxazole and sulfadiazine) in imported *Pangasius* catfish products in Thailand using liquid chromatography coupled with tandem mass spectrometry. Food Chem..

[33] Griboff J., Carrizo J.C., Bonansea R.I., Valdés M.E., Wunderlin D.A., Amé M.V. (2020). Multiantibiotic residues in commercial fish from Argentina. The presence of mixtures of antibiotics in edible fish, a challenge to health risk assessment. Food Chem..

[34] Barani A., Fallah A.A. (2015). Occurrence of tetracyclines, sulfonamides, fluoroquinolones and florfenicol in farmed rainbow trout in Iran. Food Agr. Immunol..

[35] Bortolotte A.R., Daniel D., Reyes F.G.R. (2021). Occurrence of antimicrobial residues in tilapia (*Oreochromis niloticus*) fillets produced in Brazil and available at the retail market. Food Res. Int..

[36] Carrizo J.C., Griboff J., Bonansea R.I., Nimptsch J., Valdés M.E., Wunderlin D.A., Amé M.V. (2021). Different antibiotic profiles in wild and farmed Chilean salmonids. Which is the main source for antibiotic in fish?. Sci. Total Environ..

[37] Vu T.H.A., Hai C.V., Ha H.Y., Tu N.H.K. (2021). Antibiotic resistance in salmonella isolated from ho chi minh city (vietnam) and difference of sulfonamide resistance gene existence in serovars. J. Pure Appl. Microbio..

[38] Kusunur A.B., Kuraganti G.K., Mogilipuri S.S., Vaiyapuri M., Narayanan S.V., Badireddy M.R. (2022). Multidrug resistance of *Escherichia coli* in fish supply chain: A preliminary investigation. J. Food Safety..

[39] Liu S., Bekele T.G., Zhao H., Cai X., Chen J. (2018). Bioaccumulation and tissue distribution of antibiotics in wild marine fish from Laizhou bay, North China. Sci. Total Environ..

[40] Han Q.F., Zhao S., Zhang X.R., Wang X.L., Song C., Wang S.G. (2020). Distribution, combined pollution and risk assessment of antibiotics in typical marine aquaculture farms surrounding the Yellow Sea, North China. Environ. Int..

[41] Ajslev T.A., Andersen C.S., Gamborg M., Sorensen T.I.A., Jess T. (2011). Childhood overweight after establishment of the gut microbiota: The role of delivery mode, pre pregnancy weight and early administration of antibiotics. Int. J. Obes..

[42] Schug T.T., Janesick A., Blumberg B., Heindel J.J. (2011). Endocrine disrupting chemicals and disease susceptibility. J. Steroid Biochem. Mol. Biol..

[43] Cox L.M., Blaser M.J. (2014). Antibiotics in early life and obesity. Nat. Rev. Endocrinol..

[44] Cox L.M., Yamanishi S., Sohn J., Alekseyenko A.V., Leung J.M., Cho I., Kim S.G., Li H., Gao Z., Mahana D. (2014). Altering the intestinal microbiota during a critical developmental window has lasting metabolic consequences. Cell.

