# Supplementary material for: Occurrence and Health Risk Assessment of Sulfonamide Antibiotics in Different Freshwater Fish in Northeast China
Source: Toxics. 2023 Oct 2;11(10):835. doi: 10.3390/toxics11100835 (PMC10610842; doi:10.3390/toxics11100835)
Supplement: Supplementary file 1 [file toxics-11-00835-s001.zip › toxics-2606376-supplementary.pdf]

toxics

MDPI

Supplementary materials

Sample preparation and instrumental analysis

The identification of 12 antibiotics was performed using an AB Sciex high-performance liquid chromatography (UPLC) system coupled with a 3200 Qtrap electrospray triple-quadrupole mass spectrometer (ESI-MS/MS). For the separation of pharmaceuticals, an Agilent Infinity Lab Poroshell 120 EC-C18 column (100 mm×2.1 mm，2.7 μm; Agilent, USA) was used. The mobile phase consisted of 0.2% (v/v) formic acid in water (solvent A) and methanol/acetonitrile (4:6, v/v) (solvent B) with a flow rate of 0.4 mL/min. The gradient elution program started with 5% B for 0.5 min, increased linearly up to 60% in 2.5 min, and then up to 90% in 1 min. The gradient was held further for 0.5 min and followed by a re-equilibration for 5.5 min to return to the initial phase (5% B).

The analytes were monitored using an electrospray ionization source operating in positive mode. The quantitation was performed using the multiple reactions monitoring mode. The MRM transitions, collision energies, and fragment voltages are listed in Table S1.

Human health risk assessment

Estimated Daily Intake (EDI), hazard quotient (HQ) and total hazard quotient (THQ) were calculated based on antibiotics concentrations in fish samples, which were used for human health risk assessment for the local people who consumed the fish.

EDI was computed as Eq. (1):

$$EDI = \frac{C \times IR}{BW} (1)$$

where C is antibiotics concentration (ng/g ww); BW is human body weight; IR is fish ingestion rate of humans. In the present study, the BW and IR of different age groups were derived from recent surveys (Ministry of Ecology and Environment, PRC, 2013; Shen et al., 2020).(Supplementary material for details) The population was divided into different age groups for health risk assessment, as follows: 2-5-year-old group; 6-17-year-old group; and ≥18-year-old group. The BW values were 17 kg, 41 kg, and 61 kg, and the IR values were 29.7 g/day, 37.8 g/day, and 30.1 g/day for the three age groups, respectively.

The hazard quotient (HQ) indicates non-carcinogenic risks. In this study, HQ was calculated according to Eq. (2) and used for human health risk assessment (USEPA, 2011):

$$HQ = \frac{EDI}{ADI} (2)$$

where EDI is daily intake rate (μg/kg bw/d), ADI is the acceptable daily intake of an antibiotic (μg/kg bw/d) (GB 31650-2019, 2019).

Another index, total HQ, is often calculated for assessment of risks from coexisting pollutants. This index was computed via summing up the HQ of 12 sulfonamides

$$THQs(\text{sulfonamides}) = HQ(\text{sulfapyridine}) + HQ(\text{sulfacetamide}) + HQ(\text{sulfamethoxazole}) (3)$$

where THQs < 1 indicates safe, and > 1 hazardous.

Table S1. Optimized UPLC-MS/MS parameters.

	MRM transition (m/z)	DP	CE
sulfapyridine	250.0~156.0*250.0~215.0	35	23/26
sulfadimethoxine	311.1~156.1*311.1~108.0	52	28/40
sulfamethoxazole	254.0~156.0*254.0~108.0	40	24/31
sulfachloropyridazine	284.9~155.9*284.9~108.2	35	21/34
sulfathiazole	256.0~156.0*256.0~108.0	40	20/31
sulfisoxazole	268.1~156.2*268.1~108.1	45	20/35
sulfadiazine	251.2~108.1*251.2~156.2	38	33/19
sulfamethazine	279.2~186.1*279.2~156.1	35	24/25

sulfamerazine	265.1~156.1*265.1~172.1	36	23/24
sulfamonomethoxine	281.1~156.0*281.1~215.2	38	23/26
sulfamethoxypyridazine	281.1~156.0*281.1~215.0	37	24/23
sulfacetamide	215.1~156.0*215.0~108.0	27	15/26
sulfamethoxazole -D4	258.1~160.3*258.1~112.3	62	21/32
sulfathiazole -D4	260.3~160.3*260.3~112.2	25	20/33

Table S2. Antibiotic Content in Harbin Fish (ng/g ww).

	Carassius auratus	*Ctenopharyngodon idellus*	*Cyprinus carpio*	*Aristichthys nobilis*	*Silurus asotus*	*Siniperca chuatsi*	*Hypophthalmichthys molitrix*	*Pelteobagrus fulvidraco*	*Phoxinus lagowskii*
SMX	3.23<br>ND-5.29	2.02<br>ND-5.69	3.73<br>ND-5.54	3.69<br>0.77-5.33	9.49<br>7.72-12.75	8.97<br>5.31-10.71	3.06<br>1.74-4.16	0.94<br>ND-2.08	2.98<br>0.77-5.47
SCP	1.23<br>ND-4.20	0.33<br>ND-1.02	0.17<br>ND-0.51	0.92<br>0.57-1.29	0.17<br>ND-0.61	0.24<br>ND-0.39	0.53<br>ND-1.00	0.82<br>ND-1.39	0.24<br>ND-0.53
SA	2.34<br>ND-6.36	4.17<br>ND-6.08	0.47<br>ND-1.86	1.02<br>ND-3.07	4.69<br>ND-6.94	3.78<br>ND-7.03	ND<br>ND	1.38<br>ND-2.50	5.53<br>4.66-6.52
SPD	0.23<br>ND-0.49	0.06<br>ND-0.25	0.03<br>ND-0.12	0.04<br>ND-0.12	0.80<br>ND-2.41	0.24<br>ND-0.95	ND<br>ND	0.24<br>0.13-0.44	0.05<br>ND-0.15
SDZ	0.80<br>ND-1.99	2.00<br>ND-5.26	ND<br>ND	0.33<br>ND-1.00	1.06<br>ND-1.94	1.85<br>ND-5.21	ND<br>ND	1.07<br>ND-1.76	0.43<br>ND-1.28
SMM	ND<br>ND	0.29<br>ND-1.15	0.21<br>ND-0.83	1.44<br>ND-2.98	0.98<br>ND-3.91	1.15<br>ND-3.89	ND<br>ND	0.13<br>ND-0.38	0.33<br>ND-0.65
SMP	ND<br>ND	0.15<br>ND-0.60	0.26<br>ND-1.03	1.69<br>ND-3.50	0.77<br>ND-3.08	0.96<br>ND-3.12	ND<br>ND	0.18<br>ND-0.53	0.24<br>ND-0.73
SDM	0.02<br>ND-0.06	0.25<br>ND-0.91	0.65<br>ND-2.58	9.61<br>5.38-12.46	0.66<br>ND-1.96	7.33<br>6.12-10.11	0.21<br>ND-0.55	0.68<br>0.39-0.92	0.73<br>0.22-1.02
SMT	1.03<br>ND-3.46	0.47<br>ND-1.63	1.12<br>ND-3.23	8.02<br>4.10-12.61	0.24<br>ND-0.64	6.12<br>4.37-8.53	0.36<br>ND-1.20	4.49<br>2.16-7.65	4.93<br>4.18-5.31
SMR	0.83<br>ND-3.30	ND<br>ND	0.20<br>ND-0.79	0.53<br>ND-1.58	0.22<br>ND-0.87	7.31<br>5.24-9.76	7.34<br>6.34-9.84	ND<br>ND	0.64<br>ND-1.91
STZ	5.32<br>4.05-8.62	0.22<br>ND-0.86	0.89<br>ND-1.67	0.75<br>ND-2.01	0.30<br>ND-0.74	8.27<br>6.27-12.20	9.45<br>6.92-10.72	0.19<br>ND-0.57	0.29<br>ND-0.46
SIA	1.47<br>0.59-2.39	2.53<br>ND-5.44	0.94<br>ND-2.48	0.88<br>ND-2.43	8.95<br>7.80-10.90	8.11<br>7.31-10.36	0.59<br>ND-1.45	0.50<br>ND-1.10	0.13<br>ND-0.38

ND: The concentrations less than the MQLs.

Table S3. Antibiotic Content in Changchun Fish (ng/g ww).

	Carassius auratus	*Ctenopharyngodon idellus*	*Cyprinus carpio*	*Aristichthys nobilis*	*Silurus asotus*	*Hypophthalmichthys molitrix*	*Pelteobagrus fulvidraco*	*Hemisalanx prognathus*
SMX	3.54<br>1.20-7.54	0.82<br>ND-2.37	1.36<br>ND-3.32	1.90<br>0.45-3.82	5.1<br>3.34-8.37	5.28<br>3.27-7.23	2.03<br>0.26-4.13	2.77<br>1.28-4.23
SCP	0.03<br>ND-0.12	1.01<br>0.17-1.31	0.53<br>ND-2.00	0.61<br>0.12-1.46	0.59<br>ND-1.77	1.04<br>0.98-1.15	0.63<br>0.58-0.73	0.57<br>ND-1.16
SA	ND<br>ND	0.27<br>ND-1.08	1.40<br>ND-3.36	ND<br>ND	1.40<br>ND-3.76	ND<br>ND	0.76<br>ND-2.27	0.38<br>ND-1.13
SPD	0.04<br>ND-0.16	ND<br>ND	0.12<br>ND-0.22	0.06<br>ND-0.17	0.16<br>ND-0.64	ND<br>ND	ND<br>ND	0.11<br>ND-0.32
SDZ	ND<br>ND	ND<br>ND	0.60<br>ND-2.40	0.20<br>ND-0.59	0.89<br>ND-2.30	ND<br>ND	0.43<br>ND-1.30	0.41<br>ND-0.73
SMM	ND<br>ND	0.43<br>ND-1.00	ND<br>ND	0.09<br>ND-0.28	0.50<br>ND-2.01	0.07<br>ND-0.21	0.34<br>ND-0.61	0.23<br>ND-0.70
SMP	ND<br>ND	0.19<br>ND-0.76	0.07<br>ND-0.28	0.21<br>ND-0.64	0.48<br>ND-1.17	0.40<br>ND-0.66	0.16<br>ND-0.49	ND<br>ND
SDM	0.05<br>ND-0.17	0.05<br>ND-0.17	ND<br>ND	ND<br>ND	0.02<br>ND-0.08	ND<br>ND	0.60<br>ND-1.39	0.57<br>0.35-0.82
SMT	5.8<br>3.10-8.38	1.92<br>ND-5.00	1.02<br>ND-3.76	0.52<br>ND-1.05	1.00<br>ND-4.01	0.48<br>0.08-1.13	2.04<br>0.63-3.48	1.80<br>0.32-4.07
SMR	8.02<br>6.57-10.30	0.49<br>ND-1.23	2.3<br>ND-6.66	0.82<br>ND-2.45	0.48<br>ND-1.91	7.80<br>5.22-10.70	ND<br>ND	ND<br>ND
STZ	0.07<br>ND-0.27	ND<br>ND	ND<br>ND	0.33<br>ND-0.98	0.38<br>ND-1.03	4.40<br>2.80-6.23	0.17<br>ND-0.34	0.71<br>ND-1.25
SIA	1.03<br>ND-3.28	0.55<br>ND-2.18	0.69<br>0.21-1.34	0.98<br>ND-1.52	2.67<br>0.30-6.50	0.74<br>0.20-1.07	1.29<br>ND-2.46	2.61<br>0.57-5.30

ND: The concentrations less than the MQLs.

Table S4. Antibiotic Content in Shenyang Fish (ng/g ww).

	Carassius auratus	*Ctenopharyngodon idellus*	*Cyprinus carpio*	*Aristichthys nobilis*	*Silurus asotus*	*Hypophthalmichthys molitrix*	*Pelteobagrus fulvidraco*	*Hemisalanx prognathus*	*Phoxinus lagowskii*
SMX	3.52<br>1.36-8.73	6.36<br>3.83-9.16	7.11<br>4.70-10.46	3.41<br>1.24-7.46	5.1<br>1.37-9.85	7.7<br>5.60-9.39	2.65<br>0.56-5.88	2.43<br>0.30-3.63	8.72<br>5.19-10.79
SCP	0.67<br>ND-1.41	1.72<br>0.34-2.77	0.31<br>ND-1.09	0.66<br>ND-1.04	0.59<br>ND-1.77	1.44<br>0.27-2.28	1.45<br>0.14-3.52	1.15<br>0.47-1.72	0.81<br>0.29-1.11
SA	1.18<br>ND-3.61	1.67<br>ND-5.65	3.46<br>ND-6.01	ND<br>ND	1.63<br>ND-3.76	2.79<br>ND-6.69	3.15<br>ND-5.12	2.25<br>ND-5.39	0.99<br>ND-1.98
SPD	0.65<br>0.28-0.94	0.15<br>ND-0.31	0.25<br>ND-1.00	ND<br>ND	0.13<br>ND-0.50	0.26<br>ND-0.79	0.35<br>ND-0.71	0.19<br>0.12-0.32	0.21<br>ND-0.63
SDZ	0.55<br>ND-2.20	0.29<br>ND-1.15	0.23<br>ND-0.90	ND<br>ND	0.88<br>ND-2.30	ND<br>ND	1.41<br>ND-2.63	0.86<br>ND-1.39	0.89<br>ND-1.54
SMM	0.15<br>ND-0.61	0.33<br>ND-0.78	0.59<br>ND-1.41	0.08<br>ND-0.25	0.50<br>ND-2.00	0.13<br>ND-0.40	1.56<br>ND-3.01	0.90<br>0.38-1.17	5.78<br>3.27-8.17
SMP	0.24<br>ND-0.97	0.40<br>ND-0.97	0.70<br>ND-2.19	ND<br>ND	0.47<br>ND-1.17	0.75<br>ND-1.38	1.07<br>0.22-2.23	0.30<br>ND-0.67	3.00<br>1.32-4.11

SDM	0.09<br>ND-0.26	0.38<br>ND-1.04	0.21<br>ND-0.40	ND<br>ND	0.02<br>ND-0.07	0.14<br>0.06-0.22	0.96<br>0.14-2.36	0.71<br>0.58-0.84	0.87<br>0.10-1.88
SMT	1.74<br>0.19-3.90	1.82<br>0.73-2.54	2.85<br>ND-5.10	1.07<br>ND-2.93	1.02<br>ND-3.50	2.64<br>1.55-3.38	4.32<br>2.51-7.61	2.83<br>1.58-4.12	3.46<br>2.78-4.64
SMR	6.51<br>4.49-7.19	0.21<br>ND-0.83	0.23<br>ND-0.92	2.17<br>ND-6.50	0.63<br>ND-2.50	ND<br>ND	0.88<br>ND-1.75	0.83<br>ND-1.27	3.53<br>1.64-6.53
STZ	0.41<br>ND-0.93	1.05<br>ND-1.97	0.80<br>ND-3.03	0.55<br>ND-1.02	0.38<br>ND-1.03	0.19<br>ND-0.32	2.46<br>ND-5.36	1.43<br>1.31-1.63	4.74<br>3.33-6.37
SIA	2.73<br>ND-5.57	4.78<br>2.31-6.24	3.47<br>0.33-6.37	0.95<br>0.30-1.37	2.63<br>ND-5.50	0.62<br>ND-1.55	3.25<br>2.17-5.39	2.32<br>0.84-3.47	2.53<br>1.38-3.50

ND: The concentrations less than the MQLs.
